# A low-protein diet exerts a beneficial effect on diabetic status and prevents diabetic nephropathy in Wistar fatty rats, an animal model of type 2 diabetes and obesity

**DOI:** 10.1186/s12986-018-0255-1

**Published:** 2018-03-02

**Authors:** Munehiro Kitada, Yoshio Ogura, Taeko Suzuki, Itaru Monno, Keizo Kanasaki, Ai Watanabe, Daisuke Koya

**Affiliations:** 1 Department of Diabetology and Endocrinology, Kanazawa Medical University, Uchinada, Ishikawa 920-0293 Japan; 2 Division of Anticipatory Molecular Food Science and Technology, Medical Research Institute, Kanazawa Medical University, Uchinada, Ishikawa Japan

**Keywords:** Low-protein diet, Diabetes, Fibroblast growth factor 21, High-molecular-weight adiponectin, Uncoupling protein 1, Diabetic nephropathy

## Abstract

**Background:**

The objective of this study is to investigate the effects of a low-protein diet (LPD) starting from a young age on diabetic status and renal injury in a rat model of type 2 diabetes and obesity.

**Methods:**

Diabetic male Wistar fatty (*fa/fa*) rats (WFRs) were fed a standard diet (23.84% protein) or an LPD (5.77% protein) for 24 weeks beginning at 6 weeks of age. We investigated the effects of the LPD on total body weight (BW); fat weight (FW); lower-limb muscle weight (MW); several measures of diabetic status, including fasting/random glucose levels, HOMA-IR and the IPITT; and renal injuries, including renal hypertrophy, albuminuria and histological changes. Additionally, autophagy and activation of mTORC1 were evaluated in the diabetic renal cortex. Furthermore, plasma FGF21 and high-molecular-weight (HMW) adiponectin levels, as well as UCP1 expression levels in brown adipose tissue (BAT), were evaluated.

**Results:**

Increases in BW and FW in WFRs were significantly reduced by the LPD, and the LPD resulted in a significant reduction of lower-limb MW in WFRs. The LPD suppressed the elevation of glucose levels in WFRs through improvement of insulin resistance. The LPD also elevated the plasma FGF21 and HMW adiponectin of WFRs, as well as UCP1 expression in the BAT of the animals. Renal hypertrophy, albuminuria, renal histological changes, and increased expression of p62 and phospho-S6 ribosomal protein (p-S6RP) were observed in WFRs compared with the values from WLRs. The LPD clearly prevented the diabetic kidneys from sustaining any damage.

**Conclusions:**

The LPD prevented the progression of diabetic status; this effect may have been associated with the reduction of FW and the elevation of plasma FGF21 and HMW adiponectin, as well as UCP1 expression in BAT, resulting in suppression of diabetic nephropathy. However, MW was decreased in rats by the consumption of an LPD from a young age; therefore, further research is needed to resolve the nutritional issue of LPD on decreasing in MW.

## Background

The number of patients who have type 2 diabetes is increasing worldwide. Type 2 diabetes is closely related to obesity, and the recent increase in the prevalence of type 2 diabetes worldwide has largely mirrored the rise in obesity. Diabetic vascular complications include microangiopathy, such as retinopathy, neuropathy and nephropathy, as well as macroangiopathy based on atherosclerosis. Among these diabetic vascular complications, diabetic nephropathy develops in 40% of patients with diabetes and remains the leading cause of end-stage renal disease (ESRD) [1]. In addition, the morbidity rate of cardiovascular disease (CVD) in patients with diabetic nephropathy is high, contributing to the mortality rate. There is a vicious cycle between chronic kidney disease (CKD), including diabetic nephropathy and CVD [2] because such patients suffer from not only diabetes but also obesity, dyslipidemia, hypertension, and other conditions that are recognized as common risk factors related to the progression of CKD and CVD. Therefore, multifactorial treatment to control the multiple risk factors and maintain cardiorenal metabolic health is recommended for the prevention of both diabetic nephropathy and CVD [3–5].

In recent decades, numerous novel agents for blood glucose, blood pressure (BP) and lipid control have been developed. However, controlling all risk factors in patients with diabetes is not easy, even if these numerous agents are used concurrently. Diet therapy is fundamentally important as one of the components of multifactorial treatment for diabetes [6]. In clinical situations, calorie restriction (CR), dietary restriction (DR) or a low-carbohydrate diet is enforced in patients with diabetes to improve hyperglycemia and obesity. On the other hand, a low-protein diet (LPD) is considered a viable treatment to prevent progression of renal injury in advanced CKD, including diabetic nephropathy. Although clinical evidence for the efficacy of LPDs against diabetic nephropathy is still insufficient [7–14], LPDs may exert renoprotection when patient adherence is good [15]. Previously, we also demonstrated that a very low-protein diet ameliorated advanced diabetic nephropathy by modulating the mTORC1 pathway and autophagy and prevented impairment of metabolic parameters including levels of blood glucose, total cholesterol (T-CHO), body weight (BW) and body fat weight in diabetic rats [16]. In addition, recent reports have shown that a low-protein/high-carbohydrate diet results in greater longevity and metabolic health than a high-protein/low-carbohydrate diet in mice fed those diets from 6 months of age [17]. Moreover, a previous report has shown that reduced dietary protein may retard the development of type 2 diabetes or insulin resistance in high-fat-diet-induced obese mice and New Zealand obese mice, possibly through induction of FGF21 [18]. However, it is still unclear whether an LPD enforced from a young age ameliorates metabolic impairments including diabetes and leads to the prevention of renal injury in type 2 diabetes. In the current study, we investigated the effects of a preventative LPD enforced from a young age on diabetic status and diabetic nephropathy in Wistar fatty (*fa*/*fa*) rats (WFRs), an animal model of type 2 diabetes and obesity.

## Methods

### Experimental animals

Male and female Wistar lean (*fa*/+) rats (WLRs) were provided by the Takeda Pharmaceutical Company Biological Institute (Osaka, Japan) [16] and maintained at Kanazawa Medical University. Diabetic male WFRs and age-matched non-diabetic male WLRs were used. At 6 weeks of age, the rats were divided into three groups: (1) WLRs fed a standard diet (STD), (2) WFRs fed an STD, and (3) WFRs fed an LPD. The STD contained 23.84 kcal% protein, 16.80 kcal% fat, 59.36 kcal% carbohydrates and 3.55 kcal/g energy (CLEA Japan, Inc., Tokyo, Japan), as shown in Table 1. The LPD contained 5.77 kcal% protein, 16.48 kcal% fat, 77.75 kcal% carbohydrates and 3.54 kcal/g energy (CLEA Japan, Inc., Tokyo, Japan), as shown in Table 1 [16]. The dietary intervention was performed for 24 weeks. The rats were maintained in temperature-controlled (23 ± 1 °C) rooms on a 12 h:12 h light-dark cycle with free access to water and their assigned chow. Food for the STD and LPD was purchased from CLEA (Japan, Inc., Tokyo, Japan). BW and blood glucose levels were measured every four weeks. Food intake was measured every week and is presented as the mean food intake per day. BW, abdominal fat weight (FW) including epididymal fat and retroperitoneal fat, lower-limb muscle weight (MW) and kidney weight were measured after the dietary intervention. The BP of conscious rats was measured at a steady state using a programmable tail-cuff sphygmomanometer (BP98-A; Softron, Tokyo, Japan) [16]. After 24 weeks of dietary intervention, individual rats were placed in metabolic cages for urine collection. The rats were anesthetized by inhalation of isoflurane, and the kidneys were subsequently removed after collection of blood samples from the left cardiac ventricle. The samples were stored at − 80 °C in 10% neutral buffered formalin until further use in subsequent experiments. The Research Center for Animal Life Science of Kanazawa Medical University approved all experiments, and all experiments were performed in accordance with the relevant guidelines and regulations.

**Table 1:** Source of nutrients

Source of nutrients	Standard diet (kcal%)	Low protein diet (kcal%)
Cornstarch	35.5	54.1
Milk caseins	24.5	5.85
Granulated sugar	20	20
Corn oil	6	6
Avicel cellulose	3	3
Powdered cellulose	2	2
α-starch	1	1
Vitamin mix	1	1
Mineral mix	7	7
Composition of vitamin mix		mg/100 g
Cornstarch		635.378
Choline chloride		300.000
Vitamin E (50%)		20.00
Inositol		15.00
para-aminobenzoic acid (PABA)		10.150
Nicotinic acid		10.150
Vitamin B12 (2%)		0.250
Calcium D-pantothenate		2.000
Vitamin A (100million IU/g)		1.2
Vitamin D3 (50million IU/g)		0.480
Vitamin B2		1.872
Vitamin B1		1.500
Vitamin B6		1.020
Biotin (2%)		0.5
Vitamin K3		0.3
Folate		0.2
Composition of mineral mix		mg/100 g
KH_2_PO_4_		1730.00
CaHPO_4_・2H_2_O		1500.00
CaCO_3_		1355.40
MgSO_4_・7H_2_O		800.00
Cornstarch		800.00
NaCl		600.00
FeC_6_H_5_O_7_・nH_2_O		190.00
MnSO_4_・5H_2_O		15.40
2ZnCO3・3Zn(OH)2・H_2_O		6.0
Ca(IO_3_)_2_		1.54
CuSO_4_・5H_2_O		1.26
CoCl_2_・6H_2_O		0.40

### Biochemical measurements

HbA_1c_ levels were measured using a DCA 2000 Analyzer (Siemens Medical Solutions Diagnostics, Tokyo, Japan) at the end of the experiment. Urinary albumin, plasma insulin, FGF21 and high-molecular-weight (HMW) adiponectin were measured using ELISA kits (urinary albumin: NEPHRAT II, Exocell, Inc., Philadelphia, PA, USA; plasma insulin: Morinaga Institute of Biological Science, Inc., Kanagawa, Japan; plasma adiponectin: SHIBAYAGI Co., Ltd., Gunma, Japan). Plasma total cholesterol (T-CHO) and triglycerides (TG) were measured using a Pureauto S TG-N kit (Sekisui Medical, Tokyo, Japan) and an L-type cholesterol H-test kit (Wako Pure Chemical Industries, Osaka, Japan). Urinary creatinine (Cr) was measured by enzymatic methods. The formula for homeostasis model assessment of HOMA-IR was (fasting plasma glucose × fasting plasma insulin)/405.

### IPGTT and IPITT

An IPGTT and an IPITT were performed after 24 weeks of intervention, as previously described [16]. In brief, for glucose tolerance tests, rats were fasted overnight for 16 h followed by intraperitoneal glucose injection (1 g/kg BW). Blood glucose was measured using tail blood collected at 0, 15, 30, 60, and 120 min after the injection. For insulin tolerance tests, rats were injected intraperitoneally with regular human insulin (Novolin R; Novo Nordisk, Clayton, NC) at 0.75 U/kg body weight after a 6-h fast, and blood glucose was measured 0, 15, 30, and 60 min later.

### Morphological analysis

The kidney sections were stained with Masson’s trichrome (MT). For the semi-quantitative evaluation of kidney fibrosis through MT staining in 10 randomly selected glomeruli or tubulo-interstitial areas per rat, the percentages of the areas stained for fibrosis were graded as follows: 0, 0 to 5% staining; 1, 5 to 25%; 2, 25 to 50%; 3, 50 to 75%; and 4, > 75% [16].

### Real-time PCR

The isolation of total RNA from the renal cortex or brown adipose tissue (BAT), cDNA synthesis and quantitative real-time PCR were performed as previously described [16]. TaqMan probes for type 3 collagen (Col3) (Product ID: Rn01437681), Cd68 (Rn01495634), interleukin-6 (Il6) (Rn01410330), C-C motif chemokine ligand 2 (Ccl2) (Rn00580555), Toll-like receptor 4 (Tlr4) (Rn00569848), kidney injury molecule-1 (Kim-1) (RN00597703) and uncoupling protein1 (Ucp1) (Rn00562126) were purchased from Thermo Fisher Scientific (Waltham, MA, USA) [16]. The analytical data were normalized to the levels of 18 s (Rn03928990) mRNA expression as an internal control.

### Western blotting

Western blotting was performed using antibodies against p62 (1:1000), β-actin (1:1000), phospho-S6 ribosomal protein (S6RP) (1:1000), and S6RP (1:1000), as previously described [16]. The anti-p62 antibody was obtained from Medical & Biological Laboratories (Nagoya, Japan). Anti-β-actin, anti-phospho-S6RP and anti-S6RP antibodies were obtained from Cell Signaling Technology (Danvers, MA, USA). Anti-UCP1 antibody was purchased from Abcam (Cambridge, MA, USA).

### Statistical analysis

The data are expressed as the mean ± standard deviation (SD). An ANOVA followed by Tukey’s multiple comparison test was used to determine the significance of differences among three or more groups, and Student’s *t*-test was used for unpaired comparisons. A *p* value of < 0.05 was considered significant.

## Results

### The LPD reduces metabolic impairments in diabetic and obese rats

**Fig. 1:**
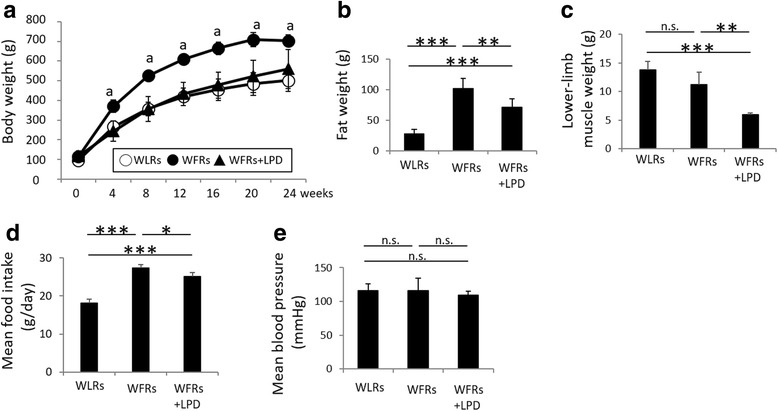
The LPD decreased the body weight, fat weight and lower-limb muscle weight of Wistar fatty rats. (**a**) Total body weight during the experimental period (*n* = 6). (**b**) Fat weight and (**c**) lower-limb muscle weight after the 24-week dietary intervention (*n* = 6). (**d**) Mean food intake during the experimental period (*n* = 6). (**e**) Mean blood pressure after the 24-week dietary intervention (*n* = 6). The data are shown as the mean ± standard deviation (SD). ^a^*p* < 0.01, STD-fed WFRs versus WLRs or LPD-fed WFRs at 4, 8, 12, 16, 20 and 24 weeks. * *p* < 0.05, **p < 0.01 and *** *p* < 0.001 for the indicated comparison. n.s.: not significant

**Fig. 2:**
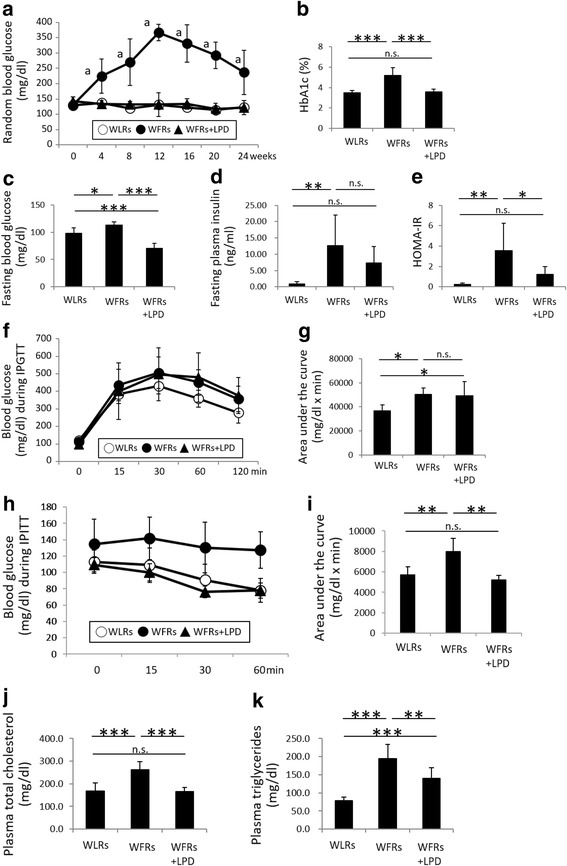
The LPD reduces metabolic impairments in Wistar fatty rats. (**a**) Random blood glucose levels during the experimental period (*n* = 6). (**b**) HbA1c levels (*n* = 6), (**c**) fasting blood glucose levels (*n* = 6), (**d**) fasting plasma insulin levels (n = 6) and (**e**) calculated HOMA-IR (*n* = 6), all measured after the 24-week dietary intervention. The blood glucose levels during the IPGTT (**f**), the IPITT (**h**), and the AUCs (**g** and **i**), which were calculated in terms of blood glucose (mg/dl) and time (minutes), for the IPGTT and IPITT after the 24-week dietary intervention (*n* = 6). (**j**) Fasting plasma T-CHO (*n* = 6) and (**k**) TG (*n* = 6) levels after the 24-week dietary intervention. The data shown are the mean ± SD. ^a^*p* < 0.001, STD-fed WFRs versus WLRs or LPD-fed WFRs at 4, 8, 12, 16 20 and 24  weeks, n.s.: not significant. * *p* < 0.05, **p < 0.01 and *** *p* < 0.001 for the indicated comparison. n.s.: not significant

The change in total BW during the experimental period is shown in Fig. 1a; the BW of STD-fed WFRs was significantly higher than that of WLRs during the experimental period. The FW of the STD-fed WFRs was significantly higher than that of the WLRs after a 24-week dietary intervention (Fig. 1b). The LPD significantly decreased the BW, FW and lower-limb MW of WFRs (Fig. 1a-c). Food intake was evaluated as the mean food intake per week. The LPD-fed WFRs ate slightly less food than the WFRs and more than the WLRs (Fig. 1d). When food intake was expressed in relation to BW, the LPD-fed WFRs tended to eat more than the STD-fed WFRs, but there was no significantly different between the two groups (STD-fed WFRs, 0.051 ± 0.003 g/g BW; LPD-fed WFRs, 0.071 ± 0.014 g/g BW). The mean BP was not significantly changed in any of the groups after the 24-week dietary intervention (Fig. 1e). The random blood glucose levels of STD-fed WFRs were higher than those of WLRs or LPD-fed WFRs during the experimental period, and the random blood glucose levels of LPD-fed WFRs did not differ from those of WLRs (Fig. 2a). In addition, the STD-fed WFRs showed significantly elevated HbA_1c_ levels compared with those of WLRs, and the LPD induced an improvement in the HbA_1c_ levels of WFRs over the 24-week dietary intervention, bringing them to the same levels found in WLRs (Fig. 2b). Fasting blood glucose levels were also higher in STD-fed WFRs than in WLRs, and the LPD reduced them compared with the levels in both WLRs and STD-fed WFRs (Fig. 2c). Fasting plasma insulin levels in STD-fed WFRs were significantly higher than those in WLRs, and LPD-fed WFRs tended towards a reduction in insulin levels compared with those of WFRs, although there was no statistically significant difference (Fig. 2d). However, the HOMA-IR of LPD-fed WFRs was significantly lower than that of the STD-fed WFRs (Fig. 2e). The blood glucose levels during the IPGTT showed no statistically significant difference between WFRs and LPD-fed WFRs; neither did the AUCs, which were calculated as blood glucose (mg/dl) x time (minutes) (Fig. 2f and g). However, the AUCs in the IPITT, which were calculated as change from blood glucose (mg/dl) x time (minutes), were significantly smaller in LPD-fed WFRs than in STD-fed WFRs (Fig. 2h and i). Plasma fasting T-CHO and TG levels were also significantly elevated in STD-fed WFRs compared with those in WLRs (Fig. 2j and k) after the 24-week dietary intervention. Increases in the T-CHO levels in WFRs were significantly reduced by the LPD intervention to the same levels found in the WLRs. However, the LPD resulted in a partial reduction in the TG levels of WFRs (Fig. 2j and k). These data indicate that the LPD ameliorated glucose elevation, insulin resistance and dyslipidemia in WFRs.

### Plasma FGF21 and HMW adiponectin levels and mRNA expression of UCP1 in BAT are elevated in LPD-fed WFRs

**Fig. 3:**
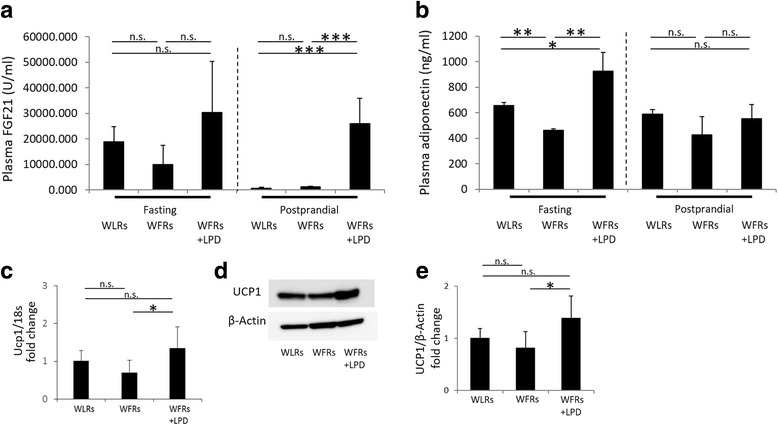
Plasma FGF21 and HMW adiponectin levels and expression of UCP1  in BAT are elevated in LPD-fed Wistar fatty rats. (**a**) Fasting or postprandial plasma FGF21, (**b**) HMW adiponectin, (**c**) and mRNA expression of Ucp1 in BAT after the 24-week dietary intervention (*n* = 3 each). (**d**) A representative photograph of immunoblotting for UCP1 in BAT is shown. (**e**) Quantitative ratios of UCP1 to β-actin in BAT after the 24-week dietary intervention (*n* = 3 each). The data are shown as the mean ± SD. * *p* < 0.05, **p < 0.01 and *** *p* < 0.001 for the indicated comparison. n.s.: not significant

Plasma FGF21 and HMW adiponectin concentrations were measured after a 16-h fast and at arbitrarily selected postprandial times (starting at 10 o’clock AM) after the 24-week dietary intervention. No differences were observed in fasting FGF21 levels among the three groups of rats (Fig. 3a). Although the plasma FGF21 levels were markedly reduced after food intake in the WLRs and STD-fed WFRs, these changes in FGF21 after food intake were not observed in the LPD-fed WFRs (Fig. 3a). In addition, plasma HMW adiponectin levels after the 16-h fast were significantly lower in WFRs than in WLRs. In LPD-fed WFRs, plasma HMW adiponectin levels showed a significantly greater increase than those of WLRs or STD-fed WFRs (Fig. 3b). In contrast, no differences were observed in the levels of plasma HMW adiponectin at the arbitrarily selected times among the 3 groups of rats (Fig. 3b). These data suggest that LPD contributes to the elevation of plasma FGF21 and HMW adiponectin. Additionally, both the mRNA expression of Ucp1 and the protein expression of UCP1 were significantly increased in the BAT of WFRs treated with the LPD compared with that of STD-fed WFRs (Fig. 3c-e).

### Changes in renal morphology, urinary albumin excretion and renal injury

**Fig. 4:**
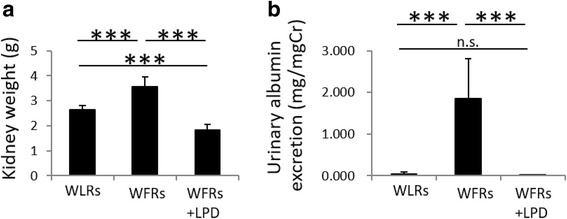
The LPD suppresses renal hypertrophy and elevation of urinary albumin excretion in Wistar fatty rats. (**a**) Kidney weight and (**b**) urinary albumin/creatinine (Cr) ratio after the 24-week dietary intervention (*n* = 6). The data are shown as the mean ± SD. *** *p* < 0.001 for the indicated comparison. n.s.: not significant

**Fig. 5:**
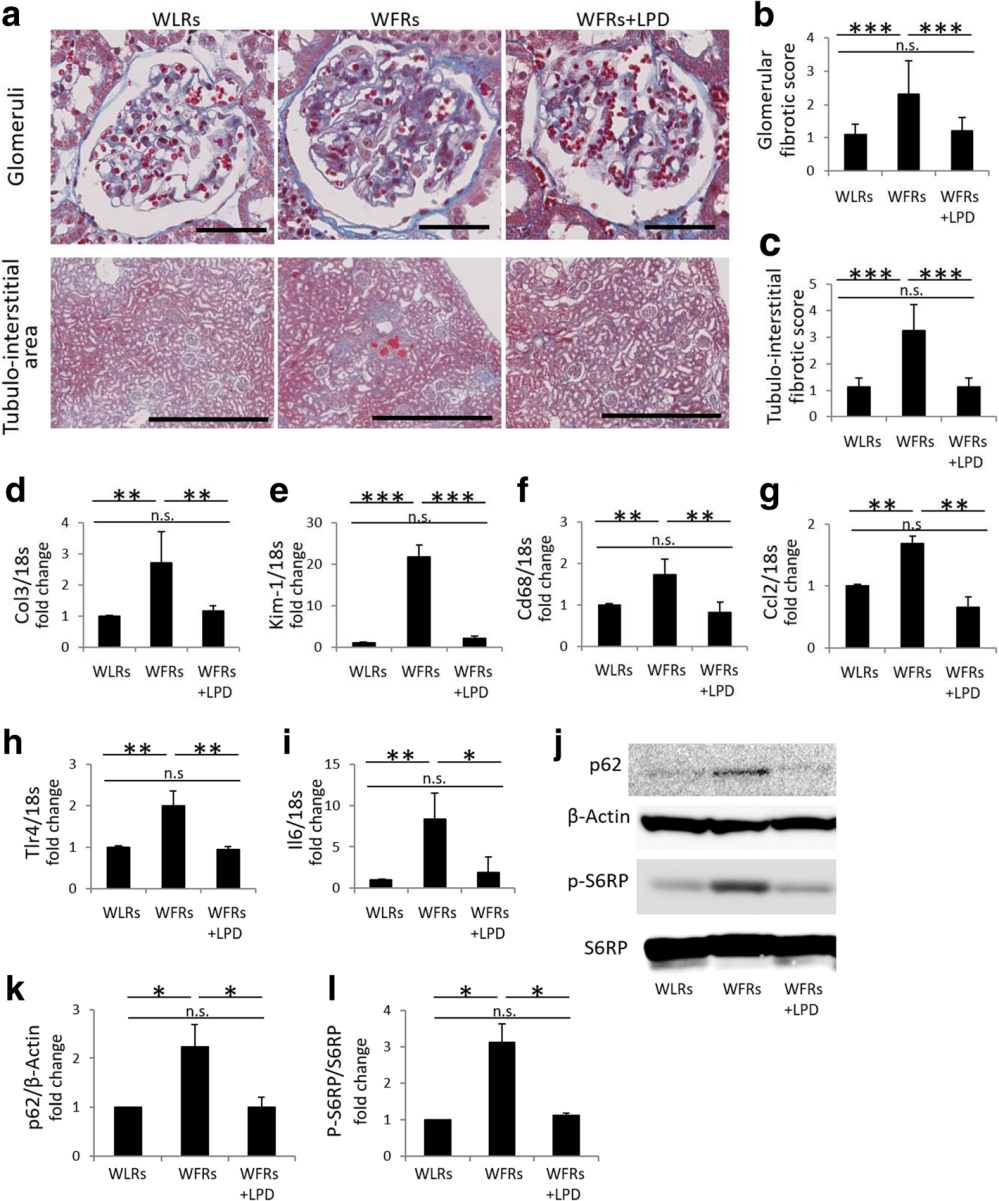
The LPD prevents renal injuries, the impairment of autophagy and the activation of mTORC1 in the kidneys of Wistar fatty rats. (**a**) Representative microphotographs of Masson’s trichrome (MT) staining of glomeruli (scale bar: 100 μm) and the tubulo-interstitial area (scale bar: 1 mm) after the 24-week dietary intervention. (**b**) Glomerular fibrotic score (*n* = 3) and (**c**) tubulo-interstitial fibrotic score (*n* = 3), assessed using MT staining after the 24-week dietary intervention. mRNA expression of Col3 (**d**), Kim-1 (**e**), Cd68 (**f**), Ccl12 (**g**), Tlr4 (**h**) and Il6 (**i**) in the renal cortex, adjusted to 18 s levels, after the 24-week dietary intervention (*n* = 6 each). (**j**) Representative photographs of immunoblotting for p62, β-actin, p-S6RP and S6RP in the renal cortex after the 24-week dietary intervention (*n* = 3). Quantitative ratios of p62 to β-actin (**k**) and p-S6RP to S6RP (**l**) (*n* = 3). Col3: type 3 collagen, Kim-1: kidney injury molecule-1, Ccl2: C-C motif chemokine ligand 2, Tlr4: Toll-like receptor 4, Il6: interleukin-6, p-S6RP: phospho-S6 ribosomal protein, S6RP: S6 ribosomal protein. The data are shown as the mean ± SD. * *p* < 0.05, **p < 0.01 and *** *p* < 0.001 for the indicated comparison. n.s.: not significant

After 24 weeks of dietary intervention, kidney weights and urinary albumin/Cr ratios were significantly higher in STD-fed WFRs than in WLRs (Fig. 4a and b). The LPD clearly ameliorated renal hypertrophy and reduced the urinary albumin levels of WFRs (Fig. 4a and b). Additionally, representative photomicrographs of MT-stained kidney sections are shown in Fig. 5a. The extent of renal fibrosis observed in glomeruli and the tubulo-interstitial area using MT staining and the mRNA expression of Col3 were shown to be higher in the kidneys of STD-fed WFRs than in the kidneys of WLRs (Fig. 5b–d). The expression of Kim-1, which is one of the markers of tubular cell damage, was also enhanced in the renal cortex of STD-fed WFRs compared with that of WLRs (Fig. 5e). In addition, the mRNA expression of inflammation-related genes including Cd68, Ccl2, Tlr4 and Il6 in renal cortex was significantly higher in STD-fed WFRs than in WLRs (Fig. 5f–i). An LPD clearly prevented all alterations including renal hypertrophy, albuminuria, fibrosis and inflammation in WFRs. These data indicate that an LPD enforced from a young age suppresses the progression of renal injuries in WFRs. In addition, immunoblotting for p62 (accumulation of p62 indicates the impairment of autophagy) was significantly enhanced in the renal cortex of WFRs compared with that of WLRs, and an LPD reduced p62 expression in the renal cortex of WFRs (Fig. 5j and k). In addition, immunoblotting for p-S6RP (a downstream target of mTORC1) was also increased in the renal cortex of STD-fed WFRs compared with that of WLRs, and an LPD decreased p-S6RP expression to levels similar to those observed in WLRs (Fig. 5j and l). These data indicate that the LPD enforced from a young age prevented the impairment of autophagy and the activation of mTORC1 in the kidneys of WFRs.

## Discussion

In this study, we demonstrated that an LPD enforced from a young age prevented the elevation of glucose levels and ameliorated insulin resistance and dyslipidemia in an animal model of type 2 diabetes and obesity, Wistar fatty rats. These improvements in diabetic status may be related to increased plasma FGF21, HMW adiponectin and UCP1 expression in the BAT of WFRs. In addition, the enforcement of an LPD from a young age prevented the progression of diabetic renal injuries including glomerular and tubulo-interstitial damage. Furthermore, the LPD resulted in the reduction of both fat and muscle weight in WFRs.

Diet therapy is critically important for the management of patients with diabetes and diabetic nephropathy [6]. An LPD is recommended for patients with advanced CKD, including those with diabetic nephropathy, and is expected to retard the decline in renal function [6]. Previously, we also demonstrated that interventional diet therapy with an LPD for 20 weeks, starting from 24 weeks of age, clearly ameliorated advanced diabetic renal injuries including tubulo-interstitial damage by restoring autophagy, which was accompanied by suppression of the mTORC1 pathway [16]. The LPD used in this study may affect glucose metabolism and obesity on the basis that it is higher in carbohydrates than an STD. Interestingly, however, the FW, HbA1c and T-CHO levels in LPD-fed WFRs were lower than those in STD-fed WFRs after 20 weeks of intervention from 24 weeks of age [16]. In the current study, data showed that an LPD enforced from a young age (6 weeks) clearly prevented the increase in glucose levels and insulin resistance in WFRs. Thereby, the LPD also prevented diabetic renal injuries including glomerular and tubulo-interstitial fibrosis, tubular cell damage and inflammation. Additionally, numerous reports including ours have shown that mTORC1 is activated in diabetic renal cells [19, 20], a process that is closely implicated in the impairment of autophagy [16]. Amino acids are required for the activation of mTORC1 [21]; therefore, the LPD, which restricts amino acid intake, prevented the activation of mTORC1 and the impairment of autophagy in the diabetic kidney in this study. However, hyperglycemia is the underlying cause of the complication of kidney injury in diabetes [22]. Therefore, in our study, an antihyperglycemic effect of the LPD intervention may help suppress diabetes-induced renal injuries, in addition to suppression of mTORC1 activation by the LPD.

The CR or DR, is the most studied dietary intervention and is known to extend life in many organisms [23], but recent evidence suggests that the balance of macronutrients, as with low-protein diets, has been shown to play a critical role, while total energy intake is less important [17, 24]. Previously, we have demonstrated that 40% DR in WFRs for 24 weeks, starting from 6 weeks of age, ameliorates diabetes-induced renal injury and partially improves glycemic control [25]. By contrast, an LPD fed to WFRs in the same time frame clearly resulted in lower glucose levels and higher insulin sensitivity than we observed in STD-fed WFRs in this study. The glucose levels were almost the same as those in the WLRs. The food intake of LPD-fed WFRs was lower than that of STD-fed WFRs. Reduction of food intake may affect BW, including fat and muscle weights, as well as diabetic status; however, the food intake of LPD-fed WFRs was only slightly decreased (by − 7.7%) compared with that pf STD-fed WFRs. Furthermore, the effects of an LPD on fat weight and glucose levels in WFRs were greater than those in 40% DR-fed WFRs, as we previously reported. Therefore, the current study showed that the LPD exerts effects that improve glucose levels and reduce fat weight, which may be independent of lower food or calorie intake in WFRs. However, when food intake was expressed in relation to BW, the LPD-fed WFRs tended to eat more than the WFRs, but there was no significant difference between two group. Solon-Biet et al. also reported that long-term investigations in ad libitum-fed mice across 25 different diets varying in macronutrient composition supported these findings, showing that late-life health and longevity were optimized not by reducing energy intake but by low-protein/high-carbohydrate diets [17]. In addition, a short-term low-protein/high-carbohydrate diet improved the levels of several markers of health including insulin, HOMA-IR, glucose tolerance and TG to levels comparable to those observed with CR, but without requiring a 40% reduction in total calorie intake [26]. Thus, an LPD may have beneficial effects against both diabetes and CKD, including diabetic nephropathy. Aging is closely related to metabolic disorders, particularly insulin resistance, and progression of renal function decline is also associated with aging [27–29]. Therefore, an LPD enforced from a young age may be a useful diet therapy for reducing aging and promoting longevity, which includes the maintenance of metabolic health and kidney function. However, the long-term effects of an LPD from a young age on aging prevention, longevity, metabolic health, and kidney function, as well as nutritional safety concerns such as sarcopenia, remain to be elucidated. In addition to the effects of the LPD itself on metabolic health, blunted weight gain in the LPD-fed WFRs may also contribute to its beneficial results.

What are the mechanisms by which an LPD improves glucose metabolism? Previous reports on studies in rodents and humans have shown that an increased systemic supply of protein or amino acids negatively affects systemic insulin action [30–32], and epidemiological studies demonstrate that dietary protein intake and type 2 diabetes incidence are positively correlated in humans [33]. One possibility is that an LPD improves glucose levels by increasing the level of FGF21. Laeger et al. demonstrated that serum levels of FGF21 specifically increase upon exposure to an LPD, regardless of overall caloric intake, in both rodents and humans [34]. FGF21 is a metabolic hormone with pleiotropic effects on the regulation of glucose and lipid homeostasis, as well as insulin sensitivity [35]. Additionally, FGF21 exerts beneficial BW-reducing effects by increasing energy expenditure [35]. In the present study, although no differences in fasting plasma FGF21 levels were observed among the three groups, non-fasting plasma FGF21 levels were not reduced in LPD-fed WFRs; however, they were decreased in STD-fed WLRs and WFRs. Thus, we propose that continuous high levels of FGF21 might be associated with lowering blood glucose and lipid levels, body weight, and fat weight in WFRs. Maida et al. showed that dietary dilution of protein/amino acids promotes improved metabolic health in type 2 diabetic mice and men, at least in part through a liver nuclear protein 1 (NUPR1)-FGF21 axis activated by select non-essential amino acid (NEAA) insufficiency [18]. However, we have no data regarding which amino acid insufficiencies are important for induction of FGF21 and the levels of NUPR1 in the liver. An LPD also enhances energy expenditure through increased sympathetic flux via β-adrenergic receptor (β-AR) signaling to BAT with consequent upregulation of UCP1 expression [36–40], as well as increased FGF21-mediated thermogenesis [34]. In addition, Laeger et al. have previously demonstrated that protein restriction increases UCP1 and promotes the browning of WAT and that these effects require FGF21 [41]. Moreover, Kwon et al. reported that UCP1 is required for FGF21-mediated improvements in glucose tolerance [42]. Our data demonstrated that an LPD-induced increase in FGF21 may be related to overexpression of UCP1 in BAT, resulting in improvement of glucose intolerance in WFRs. However, Maida et al. showed that improvement of glucose homeostasis mediated by decreased dietary protein was independent of UCP1 [18]. Although the LPD may be involved in improving glucose metabolism through the induction of FGF21, further studies are necessary whether induction of UCP1 in BAT and/or WAT is involved in the improvement of glucose metabolism in LPD-fed WFRs.

In addition to the improvement of systemic metabolic alterations including glucose metabolism, previous reports demonstrated that FGF21 protected against diabetes-induced renal injuries through anti-fibrotic, anti-inflammatory and anti-oxidative stress effects in db/db mice and in mice with diet-induced obesity and type 2 diabetes [43, 44]. Therefore, our data also indicated that the LPD might protect against diabetic nephropathy both indirectly and directly via FGF21 induction.

Another possibility for the improvement of glucose metabolism in LPD-fed WFRs is elevation of plasma HMW adiponectin. Numerous previous reports have shown that adiponectin is an adipokine that exerts a potent insulin-sensitizing effect; HMW adiponectin is the more active form of the hormone and plays a more relevant role in insulin sensitivity and in protecting against diabetes [45, 46]. Our data clearly showed that plasma HMW adiponectin levels were significantly lower in STD-fed WFRs than in WLRs and that LPD-fed WFRs exhibited higher levels of plasma adiponectin than either WLRs or WFRs, resulting in an improvement in glucose metabolism. Plasma adiponectin levels are affected by multiple factors including fat weight reduction, gender, aging, and dietary factors. In this study, fat weight reduction may be related to the elevation of plasma adiponectin in LPD-fed WFRs. Madia et al. previously reported that serum HMW adiponectin was increased with consumption of an LPD, which was also accompanied by increased serum FGF21 levels [18]. In addition, Lin et al. previously reported that adiponectin is a downstream effector of FGF21, and adiponectin couples the actions of FGF21 in local adipocytes to liver and skeletal muscle, thereby mediating the systemic effects of FGF21 on energy metabolism and insulin sensitivity [46]. However, BonDurant et al. reported that adiponectin is dispensable for the metabolic effects of FGF21 in increasing insulin sensitivity and energy expenditure [47]. In this study, there were differences in plasma FGF21 and HMW adiponectin levels between fasting and non-fasting conditions in the three groups of rats. Therefore, further study is necessary to evaluate the relationship between FGF21 and adiponectin in LPD-fed animals.

## Conclusions

An LPD enforced from a young age prevented the progression of diabetic status and the increase of fat weight, which may have been associated with increased plasma FGF21 and HMW adiponectin, as well as overexpression of UCP1 in BAT, resulting in the suppression of diabetic renal injuries in WFRs. However, the mechanism by which an LPD suppresses the incidence of diabetes has not been completely elucidated. Furthermore, there may also be some issues associated with a long-term LPD, including malnutrition or sarcopenia. Therefore, further studies are needed to resolve these points and to develop more useful dietary protocols or replacements for an LPD for metabolic health and renoprotection.

## Abbreviations

ELISA: Enzyme-linked immunosorbent assay
FGF21: Fibroblast growth factor 21
HOMA-IR: Homeostasis model assessment of insulin resistance
IPGTT: Intraperitoneal glucose tolerance test
IPITT: Intraperitoneal insulin tolerance test
mTORC1: Activation of mammalian target of rapamycin complex 1
PCR: Polymerase chain reaction
UCP1: Uncoupling protein 1
